# Effects of auditory pathway maturation on hearing loss diagnosis

**DOI:** 10.1007/s00405-026-10205-z

**Published:** 2026-04-29

**Authors:** Sabrina Savioli, P. Mancini, D. De Seta, R. Turchetta, A. Frisina, F. Y. Russo

**Affiliations:** 1 Surgery Department, San Bortolo Hospital, Vicenza, Italy; 2 Clinical and Experimental Neuroscience and Psychiatry Ph.D. program, University Sapienza of Rome, Rome, Italy; 3 Department of Sense Organs, University Sapienza of Rome, Rome, Italy

**Keywords:** Neonatal hearing screening, Auditory maturation, Pre-term infants, Tympanometry, Auditory brainstem response, Otitis media with effusion

## Abstract

**Purpose:**

The maturation of auditory pathways in newborns, particularly in pre-terms, is a crucial process influencing the accuracy and timeliness of early audiological diagnosis. The aim of this study was to investigate the effect of postnatal auditory pathway maturation on hearing loss diagnosis by comparing early and later ABR assessments in pre-term and full-term newborns.

**Methods:**

Seventy-eight newborns (156 ears) were enrolled and divided by gestational age. All underwent initial screening, clinical Auditory Brainstem Response (ABR), tympanometry, and evaluation of diagnostic timing. Statistical analyses were performed with Jamovi (v.2.3.28).

**Results:**

In full-term newborns, the initial screening significantly predicted ABR outcomes, whereas no such correlation emerged in pre-terms. However, pre-terms showed a significant improvement in ABR thresholds over time, consistent with postnatal maturational effects on auditory pathway function. Additionally, pathological tympanograms (type B) were associated with a delayed diagnosis.

**Conclusions:**

These findings stress the need for tailored protocols and extended follow-up in pre-term newborns, as well as early detection of middle ear conditions to minimize both under- and over-diagnosis.

**Supplementary Information:**

The online version contains supplementary material available at 10.1007/s00405-026-10205-z.

## Introduction

The maturation of the peripheral and central auditory pathways is a complex neurophysiological process that extends from intrauterine life to adolescence. The peripheral auditory system reaches structural maturity around the 24th-25th gestational week, whereas brainstem auditory pathways, although functional at birth, continue to mature during the first years of life. The thalamocortical auditory system follows an even more prolonged developmental trajectory, reaching full functional maturation only during adolescence [1]. These changes reflect structural and functional reorganization of the central auditory nervous system that progresses well beyond birth [2].

Auditory Brainstem Response (ABR) is considered the gold-standard tool to assess auditory function and pathway integrity in newborns. Automatic and clinical ABR, typically performed within neonatal hearing screening, evaluate transmission from the cochlea to the inferior colliculus and are crucial for the early diagnosis of hearing loss. Acoustic stimulation with tone bursts generates a sequence of waves (I-VII), whose morphology and latency provide reliable estimates of hearing thresholds. Among them, wave V is the most stable at low intensities and is routinely used as a clinical reference [3]. More broadly, auditory evoked potentials remain a cornerstone of objective auditory assessment in infants [4].

Although ABR waveforms are usually present at birth, including in pre-term infants with normal peripheral function, their latencies may reveal delayed neural conduction. Studies have consistently shown prolonged absolute latencies of waves III and V, as well as interpeak intervals, particularly the III-V segment, reflecting central brainstem transmission in pre-term compared with full-term infants [5]. Therefore, even when waveforms appear normal, the auditory brainstem of pre-term infants may still not be fully functionally mature.

This immaturity has been linked to the premature interruption of intrauterine developmental processes, affecting both cortical and subcortical auditory structures [6]. Prematurity is therefore considered a strong predictor of delayed auditory maturation and has been identified as a key variable in neonatal audiological assessment protocols [7]. Additional factors such as sex and gestational age also influence ABR outcomes [8].

## Materials and methods

All pre-term infants included in the study required admission to the neonatal intensive care unit (NICU) for a duration longer than 10 days. Exposure to known neonatal risk factors for hearing loss, including ototoxic medications, was recorded as a composite variable; however, detailed information regarding specific agents and duration of exposure was not consistently available due to the retrospective nature of the study. Exclusion criteria included syndromic subjects, confirmed genetic forms of hearing loss, maternal TORCH infections, auditory neuropathy spectrum disorder, congenital cytomegalovirus infection, and a positive family history of hearing loss.

The study was carried out in accordance with the ethical requirements of the 1964 Declaration of Helsinki, its later amendments, and the existing legislation in Italy. Ethical committee approval was not required due to the retrospective nature of the study, which utilized previously collected clinical data.

### Participants

The final sample consisted of 78 newborns (156 ears), divided into two groups based on gestational age at birth:

- *pre-term infants (study group)*: 36 infants born before the 37th week of gestation (72 ears), including 19 males and 17 females;
- *full-term infants (control group)*: 42 infants born at or after the 37th week of gestation (84 ears), including 23 males and 19 females.

Each ear was evaluated individually to allow a more accurate assessment of unilateral and bilateral hearing loss and to facilitate precise analyses of clinical variables.

### Instruments and procedures

The Level II screening was performed using the MAICO easyScreen (Interacoustics, Denmark), a portable system for automated ABR testing. Click stimuli were delivered via ear couplers, and responses were analysed through built-in algorithms providing PASS/REFER outcomes. Electrode placement and impedance checks followed manufacturer guidelines.

Otoacoustic emissions (OAEs) were routinely performed at the newborn nursery as part of the universal hearing screening program. Only infants with concordant OAEs and automated ABR findings at baseline were included. Specifically, cases showing preserved OAEs with abnormal automated or clinical ABR responses, suggestive of auditory neuropathy spectrum disorder, were systematically excluded.

Infants who received a REFER result (unilateral or bilateral) underwent subsequent clinical ABR testing (Level III) using the Lediso Eclipse system (Interacoustics, Denmark) to precisely determine hearing thresholds.

Middle ear status was systematically evaluated through tympanometry (Inventis Clarinet, Padua, Italy) in accordance with established pediatric protocols. Hearing loss classification was based on the combined interpretation of clinical ABR findings and tympanometric results. Infants presenting abnormal ABR thresholds in the presence of type B tympanograms were considered consistent with conductive hearing loss, while abnormal ABR thresholds associated with normal middle ear function (type A tympanograms) were considered consistent with sensorineural hearing loss. A detailed etiological classification was not available for all cases and was therefore not included in the analysis.

Data systematically extracted from patient records and organized into separate ear-specific databases included:

- audiological test results (TEOAE, automated ABR, clinical ABR);
- tympanogram type (type A or B).
- demographic information (sex, birth weight, gestational weeks at birth);
- age in months at the time of definitive diagnosis;
- admission to neonatal intensive care unit (NICU).

These data enabled detailed comparisons between normal and pathological ears and between pre-term and full-term infants.

Infants were evaluated in a soundproof room, during spontaneous natural sleep, without sedation.

### Statistical analyses

All statistical analyses were conducted using Jamovi software (version 2.3.28). Diagnostic delay was defined as the age in months at which a definitive audiological diagnosis was established based on the final clinical ABR assessment. Data distribution was assessed with the Shapiro-Wilk test, which indicated non-normality for all variables (*p* < 0.03). Consequently, non-parametric tests were applied. Contingency tables with Chi-square tests were used to evaluate the association between Level II screening outcomes (PASS/REFER) and final diagnoses, as well as to compare ABR confirmation patterns across groups. Wilcoxon signed-rank tests were applied to assess changes in ABR thresholds between the first and final evaluations. Mann-Whitney U tests were used to compare the age at diagnosis between pre-term and full-term infants, and to examine the effect of tympanogram type (A vs. B) on diagnostic delay. Longitudinal variations in ABR thresholds across three assessments were analysed using Friedman’s test, followed, when appropriate, by pairwise Wilcoxon comparisons. When multiple comparisons were performed, Bonferroni correction was applied according to the number of tests within each analysis. A significance threshold of *p* ≤ 0.025 was used for paired comparisons between first and final ABR thresholds within preterm and full-term groups, while a threshold of *p* ≤ 0.016 was applied for analyses involving three comparisons.

To improve readability and focus on the most clinically relevant findings, detailed statistical analyses were reported in the Supplementary Material.

## Results

Demographic and audiological characteristics are summarized in Table 1. Notable differences between the study and control groups were observed for birth weight, as well as for age at the second ABR assessment and at definitive diagnosis. The Shapiro–Wilk test indicated a non-normal distribution of the data; therefore, median, minimum, and maximum values are reported.

**Table 1:** Descriptive statistics as median, minimum, and maximum in pre-term and full-termnewborns

	Group	N	Median	Min-Max
Variable				
Weeks	Full-term	84	39	[37, 42]
	Pre-term	72	30	[23, 35]
Birth weight (g)	Full-term	84	3275	[2485, 4650]
	Pre-term	72	1250	[660, 2490]
Age at first ABR (months)	Full-term	84	3	[2, 7]
	Pre-term	72	3	[2, 6]
Age at second ABR (months)	Full-term	84	4	[4, 9]
	Pre-term	72	5	[4, 8]
Age at diagnosis (months)	Full-term	84	6	[6, 13]
	Pre-term	72	7	[6, 11]
First ABR (dB HL)	Full-term	84	40	[30, 120]
	Pre-term	72	50	[30, 120]
Second ABR (dB HL)	Full-term	84	40	[30, 120]
	Pre-term	72	40	[30, 120]
Final ABR (dB HL)	Full-term	84	35	[30, 120]
	Pre-term	72	30	[30, 110]

The hypothesis of a different maturational processes of the brainstem hearing pathways was explored by means of contingency tables and Chi-square tests, performed separately for full-term and pre-term newborns (Table 2), by means of normal outcomes (PASS/REFER) differences between the Level II and the final diagnosis of hearing loss. The comparison reached statistical significance in the full-term newborn group, whereas no significant association was observed in the pre-term group, consistent with a lower predictive value of Level II ABR assessment in the study population.

**Table 2:** Association between Level II test results (PASS/REFER) and final diagnosis of hearingloss in full-term (n=84) and pre-term (n=72) newborns. Significant values in bold

Contingency tables						
Newborns	Level II	Normal hearing (NH)	Hearing loss (HL)	χ² test	df	p-value
Full-term (*n* = 84)	PASS (*n* = 10)	10 (100%)	0 (0%)	χ²=11.4	1	< 0.001
	REFER (*n* = 74)	32 (43%)	42 (57%)			
Pre-term (*n* = 72)	PASS (*n* = 16)	10 (62%)	6 (38%)	χ²=0.468	1	0.494
	REFER (*n* = 56)	40 (71%)	16 (29%)			

To further explore the distribution of sequential ABR outcomes in the two groups in the different screening levels, a contingency table was analysed comparing full-term and pre-term newborns. The distribution of ABR confirmation patterns (HL present/absent in the two screening levels) differed significantly between the two groups (Table 3).

**Table 3:** Distribution of ABR confirmation patterns in full-term and pre-term newborns."NH–NH" indicates normal hearing confirmed at both initial and final ABR;"HL–NH" indicates initialhearing loss with subsequent normalization; "HL–HL" indicates persistent hearing loss acrossevaluations; "NH–HL" indicates delayed-onset hearing loss. Significant values in bold

Confirmation pattern	Full-term (*n* = 84)	Pre-term (*n* = 72)	χ² test	*p*-value
NH–NH	10 (11.9%)	9 (12.5%)	χ²=18.78	< 0.001
HL–NH	32 (38.1%)	40 (55.6%)		
HL–HL	42 (50%)	16 (22.2%)		
NH–HL	0 (0%)	7 (9.7%)		

To assess the progression of ABR threshold values over time, a Wilcoxon signed-rank test was conducted separately in pre-term and full-term newborns, comparing the thresholds obtained at the first and final ABR evaluations (Table 4). A significant difference in ABR thresholds was observed in the pre-term group only.

**Table 4:** Results of Wilcoxon signed-rank tests comparing initial and final ABR thresholds inpre-term and full-term infants. p value significance set after Bonferroni correction was equal0.025. Significant values in bold

Wilcoxon signed rank – full-term and pre-term infants					
		W	Z	p-value	ES
First ABR vs. Final ABR	Full term	232	1.87	0.059	*r* = 0.37
	Pre-term	1128	5.97	**< **0.001	*r* = 0.87

A Mann-Whitney U test was conducted to examine differences in age at diagnosis between full-term and pre-term neonates (Supplementary Table S1). The results indicated that pre-term infants were diagnosed at a significantly later age compared with their full-term peers. Despite reaching statistical significance, the effect size was small (*r* = 0.24), reflecting a modest but clinically relevant delay in diagnosis among pre-term neonates.

Longitudinal changes in ABR hearing thresholds were evaluated using a Friedman test, conducted separately for full-term and pre-term neonates, to determine whether significant differences occurred across repeated measures (first, second, and final diagnostic ABR) within each group. As reported in Supplementary Table S2, the Friedman test revealed significant differences across timepoints in both groups, although the Wilcoxon test showed no significant differences for full-term subjects after Bonferroni correction (Supplementary Table S3). In the pre-term group, all pairwise comparisons were statistically significant, with large effect sizes observed across timepoints.

The role of middle ear status was explored by comparing the age at diagnosis between infants with type A and type B tympanograms (Supplementary Table S4). A Mann-Whitney U test revealed a statistically significant delay in diagnosis among full-term infants with type B tympanograms, who were diagnosed 1 month later (see Table 1) than their counterparts with type A tympanograms. No significant difference was observed in the pre-term group. A larger percentage of subjects with tympanogram type b was present in full-term babies (χ²= 7.95, *p* = 0.005). When considering ears with a final diagnosis of hearing loss, conductive hearing loss (abnormal ABR with type B tympanogram) accounted for 54.8% of full-term ears and 40.9% of pre-term ears, whereas the remaining cases showed abnormal ABR with type A tympanograms (45.2% full-term; 59.1% pre-term).

To isolate the effect of auditory maturation from middle ear pathology, a subgroup analysis was conducted including only infants with type A tympanograms. Friedman test revealed a statistically significant difference in the pre-term babies only, confirming a progressive improvement in auditory thresholds (χ²=61.19, *p* < 0.001). This difference was statistically significant across all timepoints examined (Z value ranging between − 4.3 and − 5.27; *p* < 0.001; effect size > 0.7).

## Discussion

The present study investigated the impact of auditory pathway maturation on hearing loss diagnosis by comparing early screening outcomes with later clinical ABR findings in pre-term and full-term newborns. The main finding was that a significantly higher proportion of pre-term infants initially classified as REFER subsequently achieved normal hearing thresholds at final diagnosis, compared with full-term infants. This pattern supports the concept of ongoing postnatal auditory maturation in pre-term newborns and highlights the limitations of early ABR-based screening in accurately predicting permanent hearing loss in this population. However, although otoacoustic emissions were systematically assessed at baseline and cases suggestive of auditory neuropathy spectrum disorder were excluded, the lack of longitudinal otoacoustic emission measurements does not allow a complete dissociation between peripheral and central contributions to the observed ABR threshold improvements over time.

The maturation of the central auditory system, particularly of brainstem and thalamocortical structures, continues well beyond birth and can be significantly affected by prematurity. Previous studies have shown prolonged latencies of waves III and V, and lengthening of the III-V interpeak interval in pre-term newborns, reflecting delayed neural conduction in central auditory pathways [5].

The present study confirms and expands upon prior evidence, highlighting the critical influence of neural maturation on audiological diagnostic trajectories. Our findings indicate that, in full-term infants, the automated ABR (Level II) exhibits a high predictive value for the final clinical diagnosis, reflecting a more stable and mature auditory system. Conversely, in pre-term infants, this predictive value is significantly reduced, likely due to the immaturity of central auditory pathways at the time of early screening. From a clinical standpoint, a PASS/REFER output is a dichotomous screening result whose predictive value is highly dependent on both the prevalence of pathology in the tested cohort and the physiological stability of auditory responses at the time of screening. In pre-term infants, transient neurodevelopmental immaturity may increase false-positive REFER results, thereby lowering the positive predictive value of automated ABR when performed very early. In addition, because our cohort reflects a second- and third-level follow-up population enriched for pathological findings, predictive values observed in this study should not be directly extrapolated to universal newborn screening settings.

Furthermore, pre-term infants exhibited a marked improvement in auditory thresholds over time, reinforcing the hypothesis of continued postnatal maturation. This observation is consistent with longitudinal ASSR findings showing progressive maturation in pre-term compared to term infants [9]. Similar differences in screening and diagnostic follow-up between term and pre-term infants have also been reported in population-based newborn hearing screening programs. These findings reinforce the need for gestational age-tailored pathways and timely follow-up [10, 11].

Taken together, these findings indicate that the interpretation of early ABR screening results should take gestational age-related maturational variability into account. In particular, in pre-term infants, early screening outcomes may reflect transient immaturity of the auditory pathways rather than stable auditory impairment. This supports the adoption of differentiated follow-up strategies and longitudinal audiological monitoring to ensure accurate diagnosis and appropriate timing of intervention.

The distribution of diagnostic patterns (Table 3) shows significant divergence between pre-term and full-term infants. In full-term newborns, 50% had a stable diagnosis of hearing loss (HL-HL) and no cases of worsening (NH-HL), whereas in pre-term infants, only 22.2% retained the initial diagnosis, and 9.7% exhibited delayed-onset hearing loss. This result, consistent with Wada et al. [12], suggests that early ABR assessments may be less reliable in pre-term infants, reflecting an auditory system still undergoing maturation. Similar evidence has been reported in Italian cohorts, where ABR modifications were described in both full-term and pre-term newborns [13].

However, a limitation of the present study is the absence of longitudinal surveillance with otoacoustic emission measurements. The availability of such data would have enabled a more accurate interpretation of cases in which newborns exhibited a deterioration in hearing thresholds in the months following the initial hearing screening. The observed worsening of auditory thresholds may be associated with specific risk factors, including those related to prolonged hospitalization in the neonatal intensive care unit. Other possible causes underlying these circumstances include the presence of genetic variants associated with progressive forms of hearing loss. In addition, certain genetic variants confer increased susceptibility to ototoxic medications, such as aminoglycosides, which may precipitate auditory damage following exposure during the neonatal period. Another possible cause of hearing loss arising after birth is congenital infection with cytomegalovirus (CMV). However, due to the absence of widespread neonatal screening protocols for the detection of congenital CMV infection at birth, it was not possible to identify affected newborns within the study population.

In all these circumstances, the assessment of transient evoked otoacoustic emissions and distortion product otoacoustic emissions could have provided valuable information on the cochlear function to support a more accurate etiological diagnosis.

Wilcoxon and Friedman test results showed significant improvements in auditory thresholds in pre-term infants, with large effect sizes (*r* = 0.87), whereas full-term infants showed stable thresholds. This may contribute to an overestimation of hearing loss severity due to central auditory immaturity [1, 7]. This threshold evolution occurred in parallel with a later age at diagnosis in pre-term infants. It should be acknowledged that, in our cohort, the second ABR assessment was performed at a slightly later age in pre-term than in full-term infants. In clinical practice, follow-up ABR evaluations in pre-term newborns are often scheduled later to account for corrected age, medical stability, and discharge from neonatal intensive care, which may partially contribute to the observed delay in definitive diagnosis. However, the magnitude and consistency of ABR threshold improvements observed exclusively in the pre-term group, together with the high proportion of HL-to-NH transitions, suggest that delayed scheduling alone does not fully account for the observed diagnostic trajectory and support a relevant contribution of maturational processes.

These findings are consistent with Huang et al. [14], who emphasized the challenges of early diagnosis in infants presenting with chronic serous otitis media or other transient conditions influencing auditory responses. Subgroup analyses limited to infants with type A tympanograms were conducted to isolate the effect of auditory maturation from conductive factors, confirming that significant threshold improvements were observed exclusively among pre-term neonates. These results reinforce the idea that the maturation of central auditory pathways plays a decisive role in shaping the diagnostic process for pre-term infants.

The role of middle ear status in shaping the diagnostic pathway was further explored by analysing tympanometric findings. In full-term infants, the presence of a type B tympanogram was associated with a later age at diagnosis, in line with previous evidence identifying otitis media with effusion as a frequent cause of transient conductive hearing loss and diagnostic uncertainty during infancy [14, 15]. In these cases, middle ear effusion may attenuate auditory responses and complicate the interpretation of early ABR findings [16], thereby contributing to delays in definitive diagnosis.

In contrast, no statistically significant association between tympanogram type and age at diagnosis emerged in the pre-term group. This finding should be interpreted cautiously and does not necessarily indicate a lesser role of middle ear pathology in pre-term infants. Rather, it may reflect differences in clinical management, as pre-term newborns typically undergo closer audiological surveillance and more frequent follow-up assessments irrespective of middle ear status. Such structured follow-up pathways may reduce the observable impact of transient conductive conditions on diagnostic timing, potentially masking their effect in statistical analyses. Moreover, prematurity and NICU-related factors have been associated with an increased risk of chronic otitis media with effusion, which may further complicate early audiological interpretation [17]. These findings suggest that audiological follow-up protocols may benefit from being adapted to account for maturational differences between pre-term and full-term infants, thereby avoiding premature interventions such as early hearing aid fitting, with potential psychological and emotional consequences for families [11]. Early structured family-centered support programs after identification of hearing loss have been proposed to improve parental coping and engagement during follow-up [18]. The decision to provide amplification in this population is particularly complex: as noted by Frezza et al. [19], immature auditory pathways may cause transient threshold elevations, exposing very pre-term infants to the risk of overtreatment if fitted too early. Conversely, delaying rehabilitation may hinder language and cognitive development in cases of persistent impairment. Koenighofer et al. [20] further showed that delayed auditory brainstem maturation can mimic sensorineural loss in the first months of life, complicating diagnosis. Management decisions in pre-terms should therefore rely on longitudinal monitoring and repeated assessments rather than a single early ABR outcome. A tailored approach, balancing the risks of delayed intervention against those of premature device fitting, is essential to avoid both under- and over-treatment in this vulnerable group.

This study supports the role of neurological maturation in shaping the audiological diagnostic trajectory of pre-term infants, influencing both the stability of the diagnosis and the predictive value of Level II screening. Conductive pathology appears to be more relevant in full-term infants. These findings support the need for gestational-age-specific screening protocols and reinforce the importance of medium-term follow-up in pre-term infants, even in cases of early hearing loss diagnosis.

Taken together, these findings highlight the importance of integrating neurodevelopmental considerations into neonatal audiological screening and follow-up strategies.

## Conclusions

This study provides additional evidence that postnatal maturation of the auditory pathways plays a significant role in shaping the audiological diagnostic trajectory of pre-term infants. The reduced predictive value of automated ABR screening in this population, together with the longitudinal improvement in ABR thresholds, supports the hypothesis that early screening outcomes may not reliably reflect stable auditory status during the first months of life. In contrast, in full-term infants, conductive factors, particularly middle ear effusion, appear to contribute more substantially to diagnostic delay.

The results highlight the need for gestational age-specific audiological protocols. In particular, extended follow-up is warranted in pre-term infants, even in cases of initial hearing loss diagnosis, to account for ongoing central auditory development and to minimize both under and overdiagnosis. Such differentiation may enhance diagnostic precision and optimize timing for intervention.

Some limitations of the present study should be acknowledged. First, the study population reflects a second- and third-level screening cohort, resulting in a selection bias toward infants with pathological findings at initial screening. Consequently, the proportion of ears classified as REFER or diagnosed with hearing loss is higher than would be expected in a general newborn population, and the results should be interpreted within this clinical context. Second, although otoacoustic emissions were not assessed longitudinally, all included infants showed concordant otoacoustic emission and automated ABR results at initial screening, and cases suggestive of auditory neuropathy spectrum disorder were excluded. While otoacoustic emissions at baseline and the exclusion of auditory neuropathy spectrum disorder reduce the likelihood that the observed threshold improvements are attributable to peripheral dysfunction alone, the absence of longitudinal otoacoustic emission data prevents a definitive separation of peripheral and central maturational effects. Third, detailed neonatal course data were not consistently available, including the type, dosage and duration of potentially ototoxic medications and other NICU-related exposures. Therefore, we could not quantify exposure rates or adjust the analyses for these factors, which may have influenced ABR outcomes and the diagnostic trajectory.

Despite these limitations, the findings support a refinement of current neonatal hearing screening strategies, particularly in vulnerable populations such as pre-term infants, for whom developmental variability remains a critical factor. Incorporating gestational age into follow-up planning and emphasizing longitudinal assessment may help avoid premature interventions while ensuring timely identification of persistent hearing impairment.

## Supplementary information

Below is the link to the electronic supplementary material.

Supplementary Material 1

## Data Availability

The datasets generated and analysed during the current study are available from the corresponding author on reasonable request.
