# Supplementary material for: Effects of auditory pathway maturation on hearing loss diagnosis
Source: Eur Arch Otorhinolaryngol. 2026 Apr 29;283(7):4357–64. doi: 10.1007/s00405-026-10205-z (PMC13388375; doi:10.1007/s00405-026-10205-z)
Supplement: Supplementary file 1 — Supplementary Material 1 [file 405_2026_10205_MOESM1_ESM.docx]

| **Independent Samples – FULL-TERM VS PRE-TERM INFANTS** | | | | | | |  |
| --- | --- | --- | --- | --- | --- | --- | --- |
|  | **Group** | **N** | **Median** | **Min-Max** | **U** | **p-value** | **ES** |
| *Diagnosis age (months)* | Full-term | 84 | 6.00 | [6, 13] | **2298** | **0.006** | **0.24** |
|  | Pre-term | 72 | 7.00 | [6, 11] |  |  |  |

*Supplementary Table 1. Results of the Mann-Whitney U test comparing the age at diagnosis between full-term and pre-term newborns. Significant values in bold.*

| **Group** | **χ²** | **df** | **p-value** |
| --- | --- | --- | --- |
| *Pre-term (n = 72)* | **78.74** | **2** | **< .001** |
| *Full-term (n = 84)* | **11.61** | **2** | **0.003** |

*Supplementary Table 2. Friedman test results comparing ABR thresholds at three timepoints (ABR₁, ABR₂, and final diagnosis) in full-term and pre-term infants. p value significance set at 0.016 after Bonferroni correction. Significant values in bold.*

| **Group** | **Comparison** | **Z** | **p-value** | **Effect size (r)** |
| --- | --- | --- | --- | --- |
| *Pre-term (n = 60)* | ABR_1 vs ABR_2 | **–4.83** | **< .001** |  |
|  | ABR_1 vs ABR_diagnosis | **–6.02** | **< .001** |  |
|  | ABR_2 vs ABR_diagnosis | **–5.17** | **< .001** |  |
| *Full-term (n = 53)* | ABR_1 vs ABR_2 | –0.82 | 0.4 |  |
|  | ABR_1 vs ABR_diagnosis | –1.90 | 0.05 |  |
|  | ABR_2 vs ABR_diagnosis | –1.94 | 0.05 |  |

*Supplementary Table 3. Pairwise comparisons using Wilcoxon signed-rank tests.* *P value significance set at 0.016 after Bonferroni correction. Significant values in bold.*

| **Pre-term** | | | | | | |  |
| --- | --- | --- | --- | --- | --- | --- | --- |
|  | **Group** | **N** | **Median** | **Min-Max** | **Z** | **p-value** | **ES** |
| *Diagnosis age (months)* | Type A | 60 | 7 | [6, 11] | -1.55 | 0.122 | 0.275 |
|  | Type B | 12 | 8.00 | [6, 11] |  |  |  |
| **Full-term** | | | | | | |  |
|  | **Group** | **N** | **Median** | **Min-Max** | **Z** | **p-value** | **ES** |
| *Diagnosis age (months)* | Type A | 53 | 6 | [6, 9] | **-5.9** | **<.001** | **0.676** |
|  | Type B | 31 | 8 | [6, 13] |  |  |  |

*Supplementary Table 4. Results of the Mann-Whitney U test comparing the age at diagnosis between newborns with tympanogram type A and type B in both groups. P value significance set at 0.025 after Bonferroni correction. Significant values in bold.*
